# S222. CLINICAL UTILITY OF PHARMACOGENETIC TESTING IN SCHIZOPHRENIA TREATMENT

**DOI:** 10.1093/schbul/sby018.1009

**Published:** 2018-04-01

**Authors:** Daniel Mueller, Arun Tiwari, Clement Zai, James Kennedy

**Affiliations:** 1 University of Toronto; 2 Centre for Addiction and Mental Health; 3 CAMH, University of Toronto

## Abstract

**Background:**

Antipsychotics (APs), antidepressants (ADs) and mood stabilizers are essential components in treatment of most psychiatric disorders and in particular in schizophrenia. Unfortunately, among the various compounds which have been developed, lengthy trials are often required before the optimum medication treatment is found, i.e. with most significant symptom alleviation and minimal side effects. Thus, predictive factors would thus be extremely beneficial in clinical practice. The underlying reasons for this large inter-individual

variability in terms of treatment response are not fully understood. Important factors that influence drug dose, response and side effects include age, gender, patient compliance, constellation of symptoms, co-morbidity, and to a large extent genetic factors.

**Methods:**

Methods follow two strategic concepts, i.e. 1) review of the literature and review of the clinical utility of using genetic information preemptively and 2) results of own studies evaluating treatment outcome in psychiatric care after genetic information (e.g., CYP2D6 and CYP2C19) was provided to more than 350 physicians.

**Results:**

There is growing consensus among expert that genetic testing to optimize medication treatment in psychiatry meets criteria for clinical utility. However, utility remains restricted to specific gene-drug pairs and multi-gene test require further validation.

Our own research has shown that variation in genes involved in the metabolism of psychotropic drugs (pharmacokinetics) and genes encoding drug targets, such as brain receptors (pharmacodynamics) are associated with plasma drug levels, treatment response, and side effects (e.g., antipsychotic-induced weight gain). In addition, our genome-wide analyses have revealed associations with clinical outcome to antipsychotics or antidepressants and markers in neurotrophins, cell-signaling and inflammatory pathways.

With respect to our preemptive genetic testing program in more than 10,000 patients, we received supportive responses from physicians who enrolled patients in our study. Notably, while the vast majority of patients reported improvement in patient outcome, only two physicians indicated that their patient’s symptoms has slightly worsened after they had used the pharmacogenetic report to guide treatment.

**Discussion:**

There is emerging evidence that preemptive genetic testing for numerous gene & psychiatric-drug pairs has reached levels for clinical utility which includes validation of analytical and clinical validity. Genetic testing has become readily available but however clinicians and patients are poorly prepared to this new emerging field and proper education is of utmost importance. This presentation will review the level of evidence for ‘actionable’ gene-drug pairs in psychiatry in addition to present novel genomic findings and reports from our ongoing genetic testing experiences.

